# ¡ I see presenters in my house !

**DOI:** 10.1192/j.eurpsy.2021.1100

**Published:** 2021-08-13

**Authors:** M. Queipo De Llano, E. Rodríguez Vázquez, C. Capella Meseguer, J. Gonçalves Cerejeira, I. Santos Carrasco, G. Guerra Valera, A. Gonzaga Ramírez, C. Vallecillo Adame, C. De Andrés Lobo, T. Jiménez Aparicio

**Affiliations:** Psichiatry, hospital clinico universitario de Valladolid, VALLADOLID, Spain

**Keywords:** Ophthalmologists, Charles Bonnet, visual hallucinations, visual pathway

## Abstract

**Introduction:**

Charles Bonnet syndrome (CBS) is characterized by the presence of visual hallucinations, generally complex, which occurs in patients with alterations in the visual pathway. The majority of affected patients are elderly. It appears in 15% of people with visual loss, predominantly in the 80-year-old female gender.

**Objectives:**

To present a clinical case of a patient with visual hallucinations and a possible diagnosis of Charles Bonnet syndrome. Highlight the importance of an adequate differential diagnosis.

**Methods:**

Bibliographic review of the treatment and diagnosis of CBS, from articles published in the last 5 years in Pubmed.

**Results:**

Woman, 80 years old. No ophthalmological history except those associated with advanced age. She goes to the emergency room due to the presence of visual hallucinosis, in the form of “television presenters” of whom she makes partial criticism, being aware most of the time of their unreality. Hallucinations are not accompanied by anxiety or significant affective repercussions. Discarded delirium, intoxication by substances or drugs that cause the condition. Currently under follow-up to rule out other causes.

**Conclusions:**

The diagnosis of SCB requires a multidisciplinary approach between neurologists, psychiatrists and ophthalmologists in order to avoid erroneous diagnoses. The differential diagnosis should be made with pathologies such as Lewy body dementia, Parkinson’s disease, delirium, substance intoxication, migraine aura, and metabolic encephalopathy, among others. It is important to involve the family in the treatment of the syndrome to reinforce the recognition of the unreality of these hallucinations in the patients. Antipsychotic treatment can be effective only if the condition is extremely distressing.

